# Risk Prediction Model for Elderly Differentiated Thyroid Cancer Based on Combined Sleep Quality Assessment and Multimodal Ultrasound

**DOI:** 10.1002/edm2.70073

**Published:** 2025-06-27

**Authors:** Xudan Lou, Na Yi, Yingchun Liu, Yuanyuan Xu, Jieyuzhen Qiu, Xiaoming Tao, Zhijun Bao

**Affiliations:** ^1^ Department of Endocrinology Huadong Hospital Affiliated to Fudan University Shanghai People's Republic of China; ^2^ Department of Ultrasound Medicine Huadong Hospital Affiliated to Fudan University Shanghai People's Republic of China; ^3^ Department of Geriatrics Huadong Hospital Affiliated to Fudan University Shanghai People's Republic of China; ^4^ The Shanghai Key Laboratory of Clinical Geriatric Medicine Shanghai People's Republic of China; ^5^ Research Center on Aging and Medicine Fudan University Shanghai People's Republic of China

**Keywords:** multimodal ultrasound, prediction model, sleep quality, thyroid cancer

## Abstract

**Objective:**

To explore the differential diagnosis for benign and malignant thyroid nodules and the diagnostic value of sleep quality, to construct and validate a risk prediction model, providing the basis for clinical treatment decision for elderly thyroid cancer.

**Methods:**

Clinical data, Pittsburgh Sleep Quality Index (PSQI), and multimodal ultrasound were collected from elderly patients undergoing fine needle aspiration biopsy or thyroid surgery in our department of endocrinology and general surgery. Postoperative pathological results served as the gold standard, binary logistic regression identified significant risk factors, and the receiver‐operating characteristic (ROC) curves were plotted to construct and validate the prediction model.

**Results:**

Among 763 enrolled patients (566 benign and 197 malignant), multivariate analysis revealed independent risk factors: TPOAB positive, daytime dysfunction, PSQI > 7, irregular nodule shape, calcification, blood flow, high elasticity scores, and low contrast enhancement. The area under the curve (AUC) for the combined model was 0.860, significantly higher than models using multimodal ultrasound alone (AUC = 0.824) or multimodal ultrasound with TPOAB (AUC = 0.831), *p* < 0.05. The nomogram‐based prediction model demonstrated excellent discrimination, calibration, and clinical utility in internal and external validation.

**Conclusions:**

Integrating sleep quality assessment with multimodal ultrasound assisted in the differentiation of thyroid nodules in the elderly, thus may improve the preoperative diagnostic levels. Risk prediction model in a nomogram format provided an intuitive and reliable tool for clinical decision‐making.

## Graphical abstract

Sleep disorders may promote the occurrence of tumours, disrupt the rhythm, and delay the expression time of cancer‐related genes, make susceptible individuals more prone to DNA damage, and reduce the efficiency of cell repair as well. Therefore, we evaluated the individualized risk of participants through clinical data, sleep quality assessment, and multimodal ultrasound. Then, we constructed an economical, convenient, and operable prediction model to guide FNA.

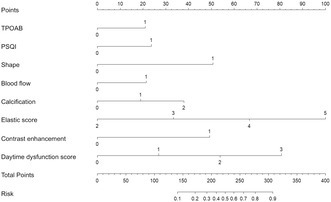

## 1 Introduction

According to the National Cancer Center, the mortality and disability‐adjusted life years (DALY) of thyroid cancer reached the peak in the elderly. From 2006 to 2016, the incidence and mortality rates of thyroid cancer in elderly women in China have both significantly increased [1]. Although there were numerous clinical differentiation methods for thyroid cancer, none of them can predict the nature of nodules alone. Previously, thyroid ultrasound imaging often followed the 2011 Kwak criteria and the 2015 ATA guidelines, but the classification system did not match the current medical situation in China. In 2020, the Chinese‐TIRADS (C‐TIRADS) based on a counting method was developed [2]. However, our preliminary research results showed the positive rate of C‐TIRADS guidance was 50.3% for fine‐needle aspiration (FNA) [3]. Even FNA has inherent problems with undiagnosed rates and high false negative rates. Surgery was performed on patients with unclear or suspected malignancy, and pathology confirmed only 15%–30% to be malignant, indicating that many patients have undergone unnecessary surgery [4].

Non‐selective biopsies of newly developed nodules may lead to a harmful epidemic in the diagnosis of thyroid cancer, while excessively conservative biopsies may result in missed diagnosis [5]. New evidence supported the use of risk assessment to select patients for FNA according to clinical data, lifestyle factors, ultrasound characteristics, and other comprehensive analyses to determine individualised risks and guide subsequent treatment [6, 7]. Of note, a large prospective research followed 140,000 women for an average of 11 years, showed that postmenopausal non‐obese women with higher insomnia scores had an increased risk of thyroid cancer [8]. Therefore, the adverse consequences of sleep interruption have attracted widespread attention and debate. Sleep disorders may promote the occurrence of tumours, disrupt the rhythm and delay the expression time of cancer‐related genes, make susceptible individuals more prone to DNA damage, as well as reduce the efficiency of cell repair [9]. Circadian rhythm disorders caused by jet lag, shift work, or sleep disorders, were all known risk factors for cancer [10].

Recently, the relationship between dysfunction of the circadian clock mechanism and thyroid cancer has been proposed. Rhythm disorders can alter the function of the HPA axis, potentially leading to peripheral clock disturbance and the occurrence of thyroid cancer [11]. Our previous research indicated that poor sleep quality (PSQI > 7), prolonged sleep latency, and daytime dysfunction were independent risk factors for elderly differentiated thyroid cancer. In this study, clinical data, sleep quality scores, and multimodal ultrasound were collected from patients scheduled for thyroid fine‐needle aspiration or thyroid surgery in our endocrinology and general surgery departments. Postoperative pathological diagnoses were used as the gold standard, and significant indicators were selected using binary logistic regression. A risk prediction model was constructed and validated using ROC curve analysis to explore methods for differentiating between benign and malignant nodules and to assess the diagnostic value of sleep quality. The results aimed to provide guidance for clinical decision‐making in the diagnosis and treatment of elderly thyroid cancer.

## 2 Material and Methods

### 2.1 Patient Enrollment and Study Protocol

According to the sample size calculation formula of the prediction model published in BMJ in 2020 [12], 763 patients scheduled for thyroid fine‐needle aspiration or thyroid surgery in our endocrinology and general surgery departments, aged ≥ 60 years, were enrolled. The exclusion criteria included the following: (a) patients with neurological disorders or severe mental illness; (b) patients with obstructive sleep apnea syndrome or irritable bowel syndrome; (c) patients with periodic limb movement disorder or restless legs syndrome; (d) patients with severe cardiovascular disease, malignant tumours, or liver and kidney dysfunction; (e) patients with a history of head and neck trauma or radiation therapy; (f) patients taking long‐term sedatives, melatonin, or psychotropic drugs; (g) patients taking medications such as amiodarone, glucocorticoids, or somatostatin that affect thyroid function; (h) patients undergoing cognitive behavioural therapy or participating in other clinical studies; (i) patients with thyroid dysfunction and those who refuse to cooperate.

This study was approved by the Ethics Committee of Huadong Hospital Affiliated to Fudan University, Shanghai, China (Ethics Number: 2018K065).

Written informed consent was obtained from all subjects prior to the study.

### 2.2 Clinical Data Collection

Clinical data were collected from elderly patients with thyroid nodules who were scheduled for fine‐needle aspiration (FNA) or thyroid surgery in our hospital, including gender, age, BMI, duration of thyroid nodule, systolic and diastolic blood pressure (measured in a sitting position, average of 2 measurements), educational level, occupation status, smoking and alcohol history, family history of thyroid cancer, as well as liver and kidney function, blood glucose, lipid profile, glycosylated haemoglobin, uric acid, thyroid function, and autoantibodies.

### 2.3 Pittsburgh Sleep Quality Index

The scale was used to evaluate sleep status over the past month and consisted of seven components: sleep quality, sleep latency, sleep duration, sleep efficiency, sleep disorder, sleep medication, and daytime dysfunction. The score range for each question was 0–3 points, with a total score of 0–21 points. The higher the score, the worse the sleep quality, PSQI > 7 defined as poor sleep quality [13], with sensitivity and specificity of 0.983 and 0.902, respectively.

### 2.4 Thyroid Nodule Evaluation

Multimodal ultrasonography referred to the combined use of two or more ultrasound examination methods for making certification and recognition more accurate. Elastography [14] and contrast‐enhanced ultrasound [15] have certain value in the diagnosis of thyroid nodules, but usually required comprehensive interpretation with morphological features of the nodules. Thus, the evaluation was performed through high‐resolution ultrasonography, elastography and contrast‐enhanced ultrasound by the same experienced ultrasound physician.

### 2.5 Model Construction and Validation

The risk prediction model was established based on the coefficients and relevant relationships, and presented in the form of a nomogram. The evaluation of the model was using ROC curves, calibration curves, and clinical decision curves. To test the reproducibility of the model and prevent overfitting, internal validation was performed using the bootstrap method with 1000 resamples. Meanwhile, collected data from another hospital was used for external validation to further estimate the portability and generalisation of the model.

### 2.6 Statistical Analysis

The data were analysed using SPSS 24.0 software. For normally distributed continuous variables, *t*‐tests were used for comparisons between two groups, and the results were expressed as mean ± standard deviation. For non‐normally distributed variables, non‐parametric tests were used, and the results were expressed as median. The chi‐squared test was for the comparisons of categorical variables, and trend tests were performed for ordinal variables. Binary logistic regression was used to analyse the relationship between categorical variables and risk factors. ROC curves were constructed, and AUC comparisons were made using MedCalc software. R language (version 3.6.3) was adopted to plot the nomogram, calibration plot and clinical decision curve. *p* < 0.05 was considered statistically significant.

## 3 Results

### 3.1 Study Characteristics

**FIGURE 1:**
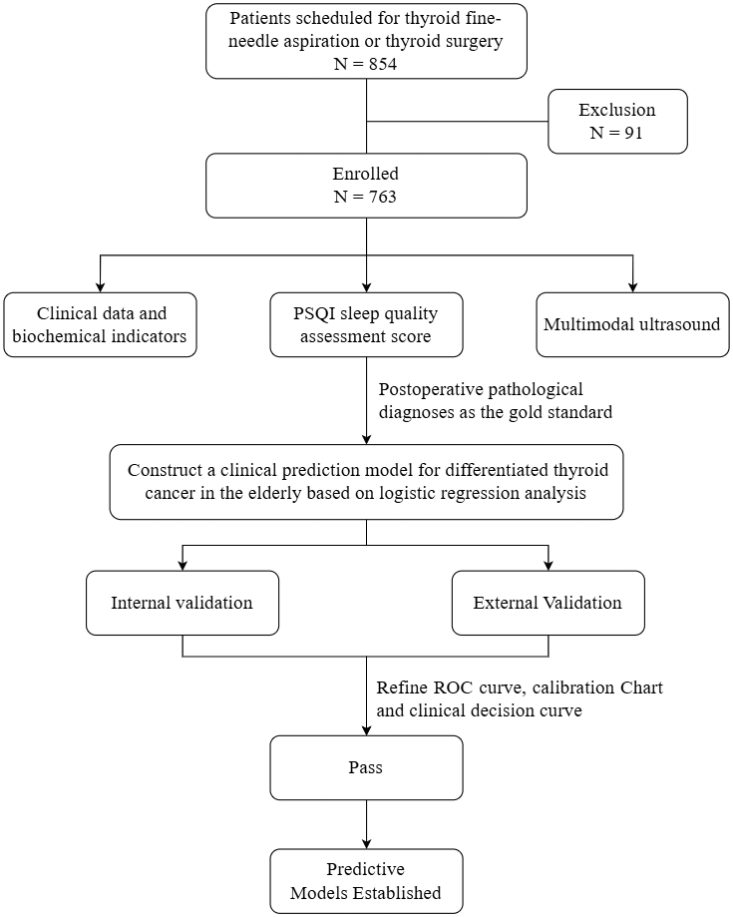
Flowchart of the study.

A total of 854 elderly patients with thyroid nodules who were scheduled for fine‐needle aspiration (FNA) and thyroid surgery in the Endocrinology and General Surgery departments of our hospital were collected. According to the inclusion and exclusion criteria, 91 patients were excluded. Finally, 763 individuals participated in this study, aged between 60 and 87 years, including 177 males and 586 females. According to the criteria for puncture and postoperative pathological diagnosis, the benign nodule group consisted of 566 people. The malignant nodule group consisted of 197 people, including 185 cases of papillary carcinoma, 12 cases of follicular carcinoma, and no medullary carcinoma or undifferentiated carcinoma. The general study flowchart was shown in Figure 1.

### 3.2 Clinical Characteristics of the Patients

Among the clinical characteristics of elderly patients with thyroid nodules, only a family history of thyroid cancer was found to be associated with malignant nodules (*p* < 0.05) (Table S1). There were no significant differences in gender, age, BMI, course of disease, blood pressure, education level, and smoking and drinking history between benign and malignant nodules (*p* > 0.05). All participants had been living in Shanghai, China for 2 years or more, with a median urinary iodine reference level of 138.4 μg/L [16].

There were no significant differences (*p* > 0.05) in biochemical indicators such as liver and kidney function, blood lipids, blood glucose, glycated haemoglobin, and blood uric acid in the comparison of benign and malignant thyroid nodules. FT3, FT4 and TSH did not show differences between the two groups. Even in the subgroup analysis of TSH, there was still no statistical significance; perhaps it was related to the thyroid function of the enrolled patients within the normal range. Only the autoantibody TPOAB had a higher malignant rate in the positive group (*p* < 0.05) (Table S2).

### 3.3 Assessment of Sleep Quality in Patients With Thyroid Nodules

There were significant differences in the comparison of sleep duration, daytime dysfunction and PSQI scores between the benign and malignant nodule groups among elderly patients (*p* < 0.05). That was to say, shorter sleep duration, poorer daytime function, and higher PSQI scores were associated with malignancy. Both groups had a PSQI score above 7, indicating sleep quality was generally poor in elderly individuals. Subgroup analysis revealed a higher incidence of malignant nodules in patients with poor sleep quality (*p* < 0.05). However, the remaining components and disease duration showed no differences between the two groups (*p* > 0.05), seen in Table 1.

**TABLE 1:** Relationship between sleep quality and thyroid nodules.

Characteristic	No.	Benign	Malignant	*p*
Sleep quality score (no./%)				
0	98 (12.84)	78 (10.22)	20 (2.62)	0.097
1	352 (46.13)	258 (33.81)	94 (12.32)	
2	244 (31.98)	173 (22.68)	71 (9.31)	
3	69 (9.05)	57 (7.47)	12 (1.57)	
Sleep latency time (no./%)				
≤ 15 min	90 (11.80)	65 (8.52)	25 (3.28)	0.057
16–30 min	277 (36.30)	221 (28.96)	56 (7.34)	
31–60 min	240 (31.45)	167 (21.89)	73 (9.57)	
≥ 60 min	156 (20.45)	113 (14.81)	43 (5.63)	
Sleep latency score (no./%)				
0	32 (4.19)	25 (3.28)	7 (0.92)	0.203
1	263 (34.47)	203 (26.61)	60 (7.86)	
2	314 (41.16)	234 (30.67)	80 (10.48)	
3	154 (20.18)	104 (13.63)	50 (6.55)	
Sleep duration score (no./%)				
0 (> 7 h)	99 (12.98)	84 (11.01)	15 (1.97)	0.028
1 (6–7 h)	255 (33.42)	191 (25.03)	64 (8.39)	
2 (5–6 h)	285 (37.35)	208 (27.26)	77 (10.09)	
3 (< 5 h)	124 (16.25)	83 (10.88)	41 (5.37)	
Sleep efficiency score (no./%)				
0	181 (23.72)	151 (19.79)	30 (3.93)	0.067
1	246 (32.24)	170 (22.28)	76 (9.96)	
2	200 (26.21)	140 (18.35)	60 (7.87)	
3	136 (17.83)	105 (13.76)	31 (4.06)	
Sleep disorder score (no./%)				
0	89 (11.66)	71 (9.31)	18 (2.36)	0.073
1	248 (32.50)	189 (24.77)	59 (7.73)	
2	287 (37.62)	212 (27.78)	75 (9.83)	
3	139 (18.22)	94 (12.32)	45 (5.90)	
Sleep medication score (no./%)				
0	262 (34.34)	225 (29.49)	37 (4.85)	0.305
1	231 (30.28)	156 (20.45)	75 (9.83)	
2	184 (24.12)	122 (15.98)	62 (8.13)	
3	86 (11.26)	63 (8.26)	23 (3.01)	
Daytime dysfunction score (no./%)				
0	136 (17.82)	125 (16.38)	11 (1.44)	0.031
1	236 (30.93)	182 (23.86)	54 (7.08)	
2	245 (32.11)	154 (20.18)	91 (11.93)	
3	146 (19.14)	105 (13.76)	41 (5.37)	
Disease duration (years)	7.13 ± 4.92	6.97 ± 4.38	7.59 ± 5.23	0.418
PSQI score	8.78 ± 2.46	8.51 ± 2.52	9.58 ± 2.08	0.000
PSQI (no./%)				
0–7	256 (33.55)	202 (26.47)	54 (7.08)	0.000
> 7	507 (66.45)	364 (47.71)	143 (18.74)	

### 3.4 Multimodal Ultrasound Evaluation of Thyroid Nodules

Characteristics of multimodal ultrasound such as the nodule number, morphology, margin, calcification, blood flow, elastography, and ultrasonic contrast between benign and malignant groups were statistically different (*p* < 0.05). Correlation analysis showed single nodular, irregular morphology, unclear margins, coarse or micro‐calcification, blood flow, higher elasticity score and low enhancement were associated with a higher risk of malignancy (*p* < 0.05). Other features had no significance in distinguishing (*p* > 0.05). Refer to Table 2 for more details.

**TABLE 2:** Relationship between multimodal ultrasound characteristics and thyroid nodules.

Characteristic	No.	Benign	Malignant	*p*
Maximum diameter (mm)	12.64 ± 9.16	12.19 ± 8.54	15.09 ± 10.80	0.059
Nodule number (no./%)				
Solitary	221 (28.97)	149 (19.53)	72 (9.44)	0.004
Multiple	542 (71.03)	417 (54.65)	125 (16.38)	
Morphology (no./%)				
Regular	535 (70.12)	446 (58.45)	89 (11.67)	0.000
Irregular	228 (29.88)	120 (15.73)	108 (14.15)	
Margin (no./%)				
Clear	578 (75.75)	461 (60.42)	117 (15.33)	0.000
Unclear	185 (24.25)	105 (13.76)	80 (10.49)	
Echo (no./%)				
Hypo echo	614 (80.47)	464 (60.81)	150 (19.66)	0.130
Non‐hypo echo	149 (19.53)	102 (13.37)	47 (6.16)	
Calcification (no./%)				
No	469 (61.47)	386 (50.59)	83 (10.88)	0.000
Coarse	190 (14.90)	120 (15.73)	70 (9.17)	
Micron	104 (13.63)	60 (7.86)	44 (5.77)	
Blood flow (no./%)				
No	531 (69.60)	422 (55.31)	109 (14.29)	0.000
Yes	232 (30.40)	144 (18.87)	88 (11.53)	
Elasticity score	3.82 ± 0.96	3.39 ± 0.93	4.32 ± 0.73	0.000
Elasticity score (no./%)				
2	52 (6.82)	52 (6.82)	0 (0)	0.000
3	305 (39.97)	266 (34.86)	39 (5.11)	
4	223 (29.23)	149 (19.53)	74 (9.70)	
5	183 (23.98)	99 (12.97)	84 (11.01)	
Ultrasonic contrast (no./%)				
High enhanced	430 (56.36)	363 (47.58)	67 (8.78)	0.035
Low enhanced	333 (43.64)	203 (26.60)	130 (17.04)	
Uniform perfusion	402 (52.69)	302 (39.58)	100 (13.11)	0.141
Uneven perfusion	361 (47.31)	264 (34.60)	97 (12.71)	

### 3.5 Logistic Regression Analysis

Considering the lower risk of outcome events as the continuous independent variable changes by one unit, this study discretized the continuous variables into multiple categorical variables for analysis. When these variables were included in a multivariate logistic regression analysis, eight independent risk factors for malignant thyroid nodules were ultimately identified, *p* < 0.05 (Table 3).

**TABLE 3:** Univariate and multivariate logistic regression.

	Single factor analysis			Multiple‐factor analysis		
	OR	95% CI	*p*	OR	95% CI	*p*
Family history of thyroid cancer	2.429	1.146–5.145	0.021	2.300	0.773–6.843	0.134
TPOAB positive	1.468	1.038–2.077	0.030	1.772	1.115–2.814	0.015
Sleep duration score	1.296	1.081–1.555	0.005	1.230	0.951–1.590	0.114
Daytime dysfunction score	1.707	1.430–2.037	0.000	1.832	1.420–2.363	0.000
PSQI score > 7	2.133	1.486–3.063	0.000	1.584	1.009–2.761	0.003
Nodule number (solitary)	1.667	1.177–2.361	0.004	1.989	0.960–3.140	0.105
Morphology (irregular)	4.459	3.149–6.313	0.000	3.601	2.231–5.812	0.000
Margin (unclear)	2.946	2.059–4.215	0.000	1.230	0.740–2.046	0.425
Calcification	2.055	1.645–2.567	0.000	1.576	1.182–2.102	0.002
Blood flow	2.358	1.666–3.337	0.000	1.700	1.081–2.673	0.022
Elasticity score	2.553	2.080–3.133	0.000	2.419	1.898–3.083	0.000
Contrast enhancement (low)	3.470	2.467–4.880	0.000	3.787	2.460–5.832	0.000

**FIGURE 2:**
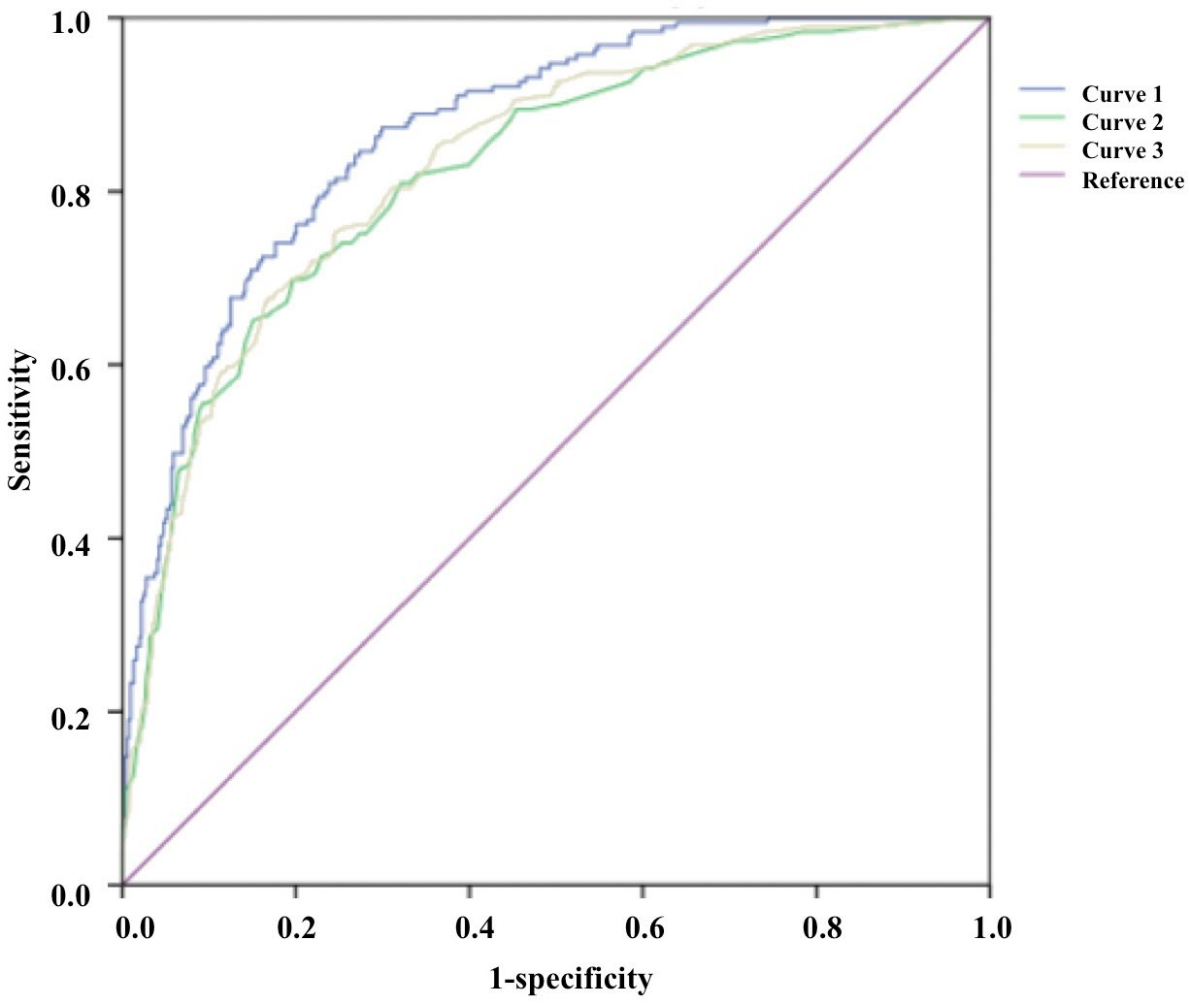
Comparison of the area under ROC curves. Curve 1: The comprehensive analysis of all independent risk factors. Curve 2: The individual multimodal ultrasound. Curve 3: The combination of multimodal ultrasound and TPOAB. Curve 1 versus Curve 2, *p* < 0.05; Curve 1 versus Curve 3, *p* < 0.05; Curve 2 versus Curve 3, *p* > 0.05.

Figure 2 revealed ROC curves for the predicted probabilities; the AUC of curve 1 was 0.860 (95% CI 0.841–0.896) for the comprehensive analysis of all independent risk factors, with a sensitivity of 82.5% and a specificity of 74.1%. AUC for the individual multimodal ultrasound (curve 2) was 0.824 (95% CI 0.790–0.858), with a sensitivity of 69.5% and a specificity of 80.5%. AUC for the combination of multimodal ultrasound and TPOAB (curve 3) was 0.831 (95% CI 0.798–0.864), with a sensitivity of 67.4% and a specificity of 83.2%. Results showed significant differences only when curve 1 was compared with curve 2 or curve 3, *p* < 0.05, indicating that the combination of sleep quality assessment can improve the diagnostic accuracy of malignant thyroid nodules in elderly patients.

### 3.6 Model Performance

**FIGURE 3:**
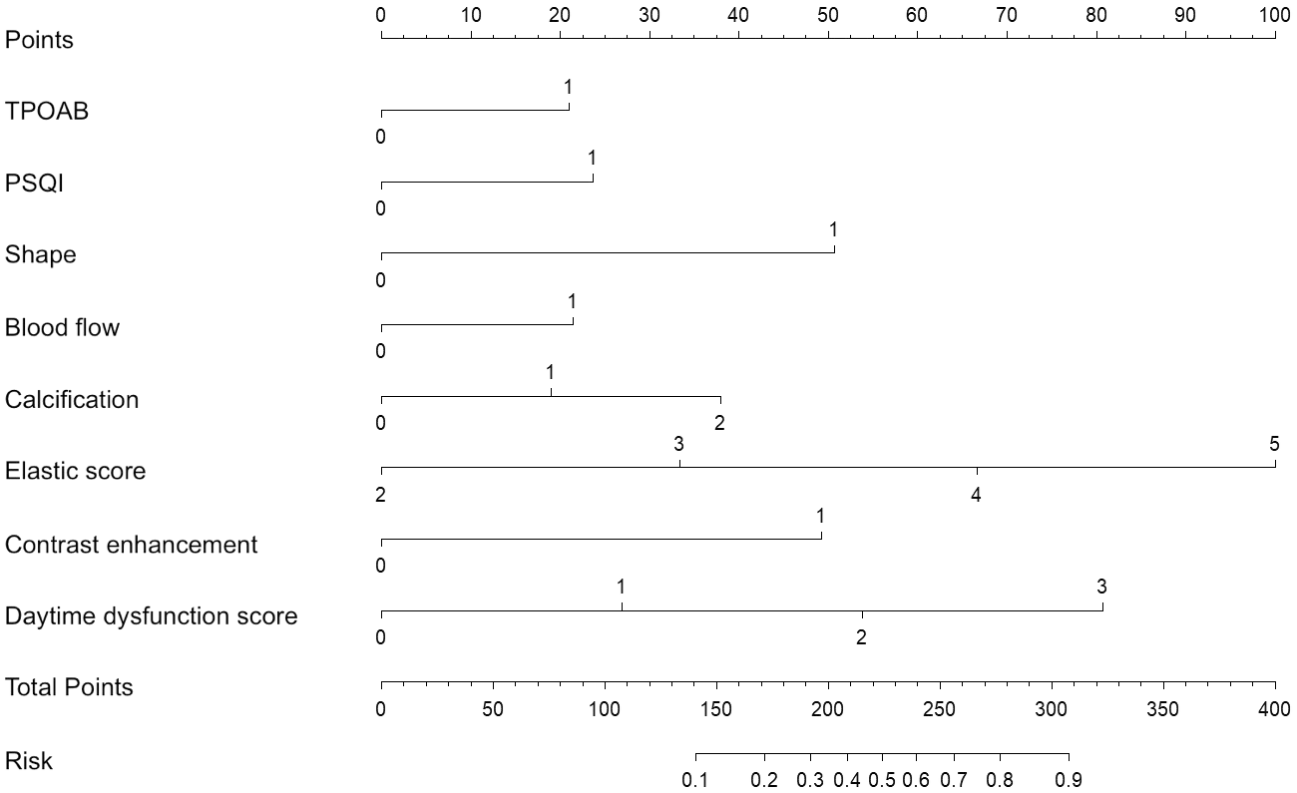
Nomogram of the risk prediction model.

Based on the results of logistic regression analysis, a risk prediction model for elderly thyroid cancer was constructed using the R language [17]. The model represents the relationships between variables in an intuitive way using a nomogram [18, 19]. The nomogram can be used as follows: draw a vertical line for each variable value, locate the variable score on the first line, and sum up all the scores to obtain the total score. Read the thyroid cancer risk probability on the total score axis vertically (Figure 3).

**FIGURE 4:**
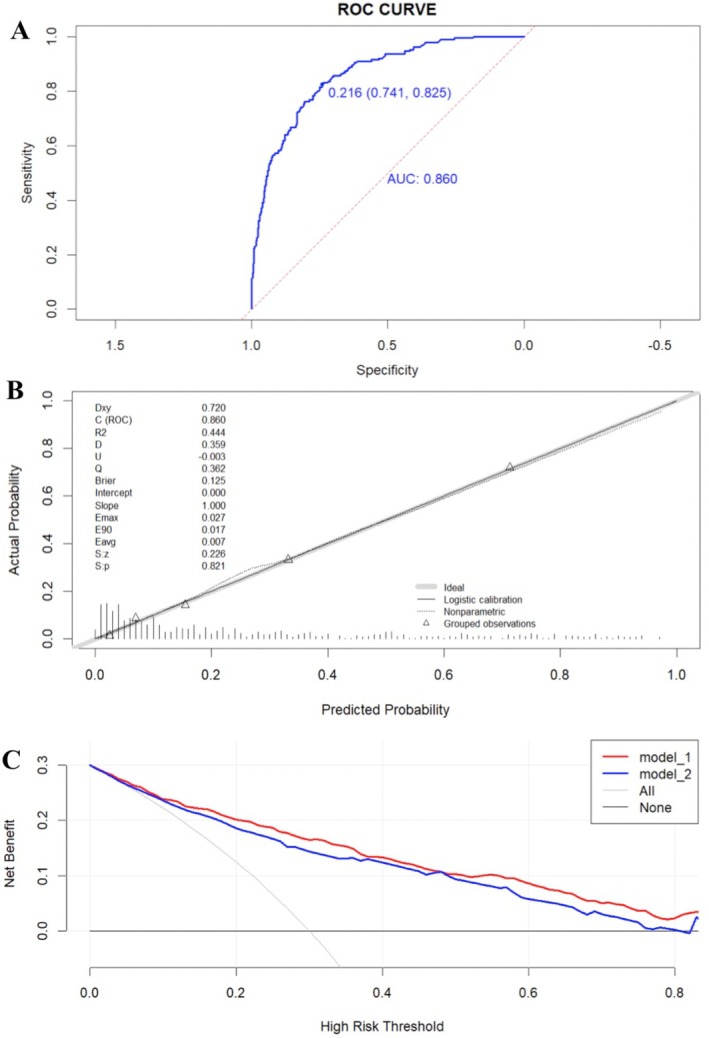
Model performance. (A) ROC curve, (B) calibration curve and (C) clinical decision curve to evaluate discrimination, calibration ability and clinical utility of the risk prediction model, respectively. Model_1: Sleep quality combined with multimodal ultrasound. Model_2: Individual multimodal ultrasound.

The accuracy of the prediction model was 0.825, and the AUC was 0.860 (Figure 4A). The calibration ability was evaluated using Hosmer‐Lemeshow goodness‐of‐fit test, result showed *X* ^2^ = 4.218, *p* = 0.821. The predicted probability was very close to the actual probability (y = x), indicating high calibration of the model (Figure 4B). A decision curve analysis (DCA) was conducted to evaluate the clinical utility (Figure 4C). The horizontal line (None) represented all samples being negative, where no interventions were performed and the net benefit was zero. The diagonal line (All) represented all samples being positive, where all individuals received interventions, and the net benefit was a negatively sloping line [20]. The curves for model_1 and model_2 were both above the two extreme curves, and model_1 had a higher net benefit within a larger threshold probability range, suggesting good clinical utility of the prediction model.

### 3.7 Verification Study

Adopting the Bootstrap method with 1000 iterations for internal validation, the Kappa statistic was used to measure the stability of the predictive model. The results showed an accuracy of 0.818 and a Kappa value of 0.486; furthermore, the C‐index was 0.804, indicating good discrimination and accuracy of the model in the internal validation.

**FIGURE 5:**
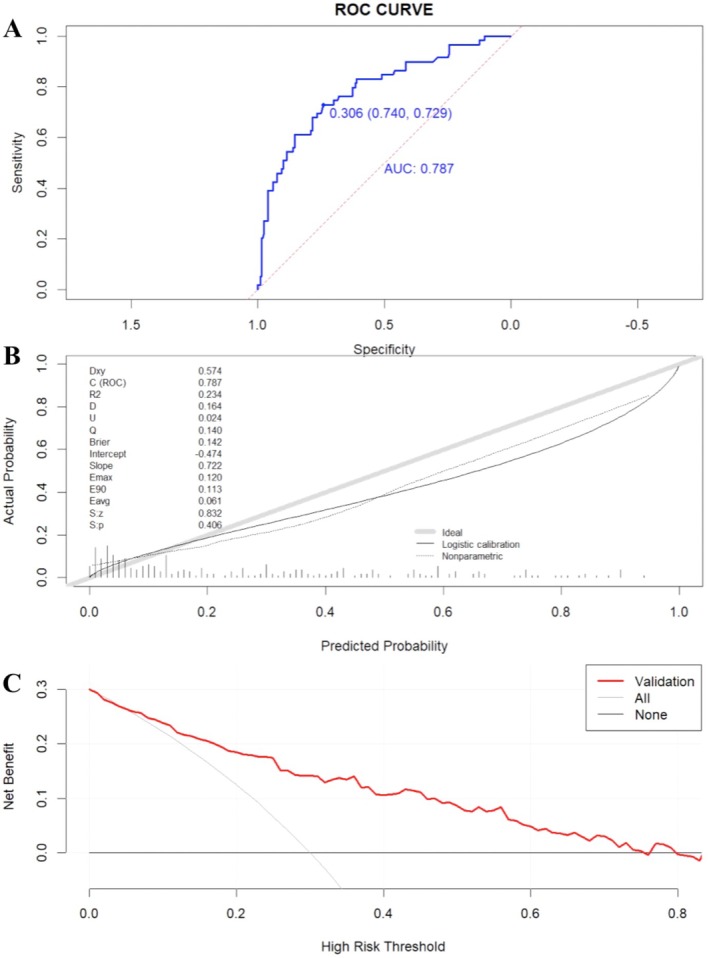
External validation of the risk prediction model. (A) ROC curve, (B) Calibration curve and (C) Clinical decision curve to evaluate the discrimination, calibration ability and clinical utility of the validation cohort, respectively.

The study used elderly patients undergoing thyroid fine needle aspiration biopsy and surgery at Shanghai North Hospital as an external validation cohort. A total of 221 patients were collected between January 2018 and January 2024; 198 patients were ultimately enrolled based on the inclusion and exclusion criteria. There were no statistical differences between the predictive variables of the validation cohort and the development cohort (Table 4). The AUC was 0.787, with a sensitivity of 72.9% and specificity of 74.0% (Figure 5A). The Hosmer‐Lemeshow test showed *X* ^2^ = 5.855, *p* = 0.406 (Figure 5B). The DCA plot indicated that the predictive model consistently outperformed the two extreme curves within the threshold probability range of 0.1–0.7 (Figure 5C). These results demonstrated that the predictive model had similar discrimination, calibration and clinical utility in the external validation.

**TABLE 4:** Clinical data, sleep quality and multimodal ultrasound features of the validation.

Characteristic	Total number	Benign	Malignant
TPOAB (no./%)			
Negative	156 (78.79)	122 (61.62)	34 (17.17)
Positive	42 (21.21)	30 (15.15)	12 (6.06)
Daytime dysfunction score (no./%)			
0	19 (9.60)	16 (8.08)	3 (1.52)
1	58 (29.29)	54 (27.27)	4 (2.02)
2	87 (43.94)	59 (29.80)	28 (14.14)
3	34 (17.17)	23 (11.62)	11 (5.55)
PSQI (no./%)			
0–7	49 (24.75)	35 (17.68)	14 (7.07)
> 7	149 (75.25)	117 (59.09)	32 (16.16)
Morphology (no./%)			
Regular	135 (68.18)	113 (57.07)	22 (11.11)
Irregular	63 (31.82)	39 (19.70)	24 (12.12)
Calcification (no./%)			
No	97 (48.99)	77 (38.89)	20 (10.10)
Coarse	66 (33.33)	49 (24.75)	17 (8.58)
Micron	35 (17.68)	26 (13.13)	9 (4.55)
Blood flow (no./%)			
No	122 (61.62)	97 (48.99)	25 (12.63)
Yes	76 (38.38)	55 (27.78)	21 (10.60)
Elasticity score (no./%)			
2	15 (7.57)	15 (7.58)	0 (0)
3	65 (32.83)	56 (28.28)	9 (4.54)
4	69 (34.85)	48 (24.24)	21 (10.61)
5	49 (24.75)	33 (16.67)	16 (8.08)
Ultrasonic Contrast (no./%)			
High enhanced	103 (52.02)	92 (46.46)	11 (5.56)
Low enhanced	95 (47.98)	60 (30.30)	35 (17.68)

## 4 Discussion

Since the 1970s, thyroid nodules have been detected in over 60% of the general population through ultrasound imaging. However, most of these nodules are benign and do not require further treatment, making accurate screening for malignant nodules a major challenge in the management of thyroid disease. FNA is the most widely used cytological examination method and can differentiate approximately 75%–80% of nodules. In a study, when both malignant tumours and suspicious malignant tumours were classified as positive, the sensitivity of FNA alone was 90.7% and the specificity was 85.2% [21], which affects the routine use of FNA as a frontline screening tool. A previous large‐sample study showed that repeated FNA of atypical lesions or follicular lesions of undetermined significance (AUS/FLUS) does not improve the detection accuracy of malignant tumours [22]. To benefit elderly patients with truly malignant nodules during surgery, improve the quality of life of the elderly population, and allocate medical resources effectively, we evaluated the individualized risk of participants through clinical data, sleep quality assessment and multimodal ultrasound, then constructed an economical, convenient and operable prediction model to guide FNA decision.

According to the research flow chart, a total of 763 patients were enrolled, 566 with benign nodules and 197 with malignant ones. All the malignant nodules were differentiated, and the malignant rate was 25.82%. Univariate analysis showed that family history, TPOAB positive, sleep duration score, daytime dysfunction score, PSQI > 7, solitary nodule, irregular shape, unclear margins, nodule calcification, blood flow, elastic score, and low contrast enhancement may be associated with an increased risk of malignant nodules. Further logistic regression analysis identified eight independent risk factors for thyroid cancer, as shown in Table 3. Patients with Hashimoto's thyroiditis (HT) usually have elevated levels of thyroid antibodies in serum, or are positive for TPOAB alone, accounting for 97% of literature reports [23]. In 1955, Dailey first proposed a close relationship between HT and papillary thyroid carcinoma (PTC) [24]. In a retrospective analysis of 8524 cases of thyroid surgery in China, it was found that the risk of PTC in HT patients was significantly increased [25], which was consistent with our research results.

Sleep deprivation prolonged exposure to night‐time light, affecting melatonin and TSH secretion, leading to circadian rhythm disruption. In 2007, the International Agency for Research on Cancer classified staying up late, including night shift work involving circadian rhythm disorders, as a Group 2A carcinogen [26]. A prospective cohort study targeting female nurses, after 26 years of follow‐up, found evidence that night shift work, extreme sleep duration, and sleep difficulties collectively affected the risk of thyroid cancer [27]. Another large‐scale prospective study of 460,000 participants showed that exposure to artificial light at night greatly increased the incidence of thyroid cancer [28]. According to clinical research, 57% of elderly people aged 60 and above in China experienced some degree of sleep disorders, with most sleeping for about 5 h and frequent waking at night. The duration of light sleep was much longer than that of deep sleep, making them a group with severe sleep disorders. A recent study used genome‐wide association studies (GWAS) published in the FinnGen and UK Biobank databases to explore the causal relationship between sleep characteristics and thyroid cancer risk based on Mendelian randomisation. The results revealed that shortened sleep time was associated with thyroid cancer [29], indicating the importance of adequate sleep in preventing thyroid cancer. These prior studies aligned with our findings linking circadian disruption to thyroid carcinogenesis.

The ultrasound risk stratification system and thyroid biopsy threshold for thyroid nodules varied in different versions of the guidelines, including C‐TIRADS, which may not reliably predict nodule properties and guide FNA. Our study used multimodal ultrasound combined with PSQI to plot ROC curves. The AUC obtained from the integrated factors was 0.860 (95% CI 0.841–0.896), with a sensitivity of 82.5% and specificity of 74.1%, higher than that of the other two curves, *p* < 0.05 (Figure 2). The results showed that sleep quality assessment and multimodal ultrasound may improve the diagnostic level of malignant nodules, providing clinical reference value for FNA. Based on the above statistical analysis, a risk prediction model for elderly thyroid cancer was constructed and presented in the form of a nomogram (Figure 3). The combined model achieved an AUC of 0.860, outperforming single‐modality approaches and showing good discrimination. The calibration curve aligned closely with ideal predictions (Hosmer‐Lemeshow, *p* = 0.821), and DCA confirmed clinical utility (Figure 4A–C). External validation yielded an AUC of 0.787, demonstrating repeatability and generalizability (Figure 5A–C).

Researchers both domestic and international have emphasized the importance of developing multivariate prediction algorithms to determine the cumulative risk of malignant tumours for this common clinical problem. Raza et al. [30] used a multivariate stepwise regression model to predict the malignancy rate of thyroid nodules in patients based on factors such as patient age, solid/nodule calcification, and FNA cytology examination. Tuttle applied Bayesian analysis modeling and found that male gender, nodules larger than 4 cm, and glandular features could be systematically integrated into clinical decision‐making, thereby reducing the probability of surgery for follicular tumour patients [31]. Alexander et al. used prospective cohorts for Bayesian classification and constructed and cross‐validated a clinically relevant prognostic assessment tool [32]. Another study constructed a multivariate logistic regression model with all ultrasound features for 1500 patients from Shanghai and Fujian, incorporating variable weights and combination patterns to predict PTC, FTC, and MTC [33]. Therefore, significant progress has been made in understanding the clinical significance of thyroid nodules and insufficient evaluation of potential harms. Various clinical models can estimate the thresholds for thyroid nodule biopsies to some extent, balancing the diagnostic value of thyroid cancer and the potential risk of missed diagnosis. However, the choice of the model needs to consider specific patients, population preferences, regional characteristics, operational conditions, etc. Predictive models that combine clinical, biochemical, and radiological features can support clinical doctors in reducing unnecessary invasive surgeries for thyroid nodule patients [34].

This study focused on constructing a prediction model for elderly thyroid cancer, highlighting the synergistic value of sleep quality assessment and multimodal ultrasound. It not only provided personalised risk of malignancy but also allowed real‐time evaluation of suspicious nodules, promoting clinical decision‐making and patient education. In cases where FNA examination was limited, such as poor patient health, difficulty in puncturing small nodules, inadequate tissue obtained for diagnosis, or uncertain diagnosis, the model also demonstrated its advantages.

## 5 Limitations

First, a major limitation of this study was the lack of prospective validation of the model, as well as the small size and limited sample of the validation cohort, which were derived from a single‐centre dataset. While we followed the recommended minimum of 10 events per predictive variable, it was necessary to validate the model in a larger patient population. Second, the malignancy rate of thyroid nodules in this study was 25.82%. The selection of patients who underwent thyroid nodule puncture or surgery inevitably led to a significantly higher proportion, which may not objectively reflect the incidence of thyroid cancer in the elderly population. Third, the questionnaire used in this study was subjective, and recall bias may exist.

## Author Contributions

Xudan Lou was the major contributor in writing the manuscript. Na Yi and Yuanyuan Xu collected the data information of the patients. Yingchun Liu was responsible for the multimodal ultrasound examination. Jieyuzhen Qiu made a statistical analysis. Xiaoming Tao and Zhijun Bao designed and funded the study. All authors read and approved the final manuscript.

## Ethics Statement

The study was carried out in accordance with The Code of Ethics of the World Medical Association (Declaration of Helsinki) and approved by the Ethics Committee of our institute.

## Conflicts of Interest

The authors declare no conflicts of interest.

## Supporting information


Tables S1–S2.

## Data Availability

The data that support the findings of this study are available from the corresponding author upon reasonable request.
