# Supplementary material for: Risk Prediction Model for Elderly Differentiated Thyroid Cancer Based on Combined Sleep Quality Assessment and Multimodal Ultrasound
Source: Endocrinol Diabetes Metab. 2025 Jun 27;8(4):e70073. doi: 10.1002/edm2.70073 (PMC12205140; doi:10.1002/edm2.70073)
Supplement: Supplementary file 1 — Tables S1–S2. [file EDM2-8-e70073-s001.docx]

**Table S1. Relationship between Clinical Characteristics of Patients and Thyroid Nodules**

| **Characteristic** | **No.** | **Benign** | | **Malignant** | ***P* value** |
| --- | --- | --- | --- | --- | --- |
| Gender (no./%) |  | |  |  | 0.433 |
| male | 177 (23.20) | | 123 (16.12) | 54 (7.08) |  |
| female | 586 (76.80) | | 443 (58.06) | 143 (18.74) |  |
| Age (years) | 67.31±5.85 | | 67.68±6.05 | 66.25±5.09 | 0.221 |
| Age (no./%) |  | |  |  | 0.103 |
| 60-69 | 533 (69.85) | | 375 (49.14) | 158 (20.71) |  |
| 70-79 | 189 (24.77) | | 155 (20.31) | 34 (4.46) |  |
| 80-87 | 41 (5.38) | | 36 (4.72) | 5 (0.66) |  |
| BMI (kg/m2) | 24.14±6.07 | | 23.99±6.57 | 24.52±4.38 | 0.314 |
| Course (years) | 3.05±4.91 | | 3.26±4.95 | 2.45±4.71 | 0.051 |
| Blood pressure (mmHg) |  | |  |  |  |
| Systolic pressure | 128.18±19.47 | | 128.41±19.96 | 127.83±18.74 | 0.808 |
| Diastolic pressure | 76.31±11.01 | | 76.10±11.27 | 76.65±10.61 | 0.682 |
| Education level (no./%) |  | |  |  | 0.356 |
| primary school | 88 (11.53) | | 64 (8.39) | 24 (3.14) |  |
| middle school | 326 (42.73) | | 236 (30.93) | 90 (11.80) |  |
| high school | 272 (35.65) | | 203 (26.61) | 69 (9.04) |  |
| college and above | 77 (10.09) | | 63 (8.26) | 14 (1.83) |  |
| smoking or drinking history (no./%) |  | |  |  | 0.448 |
| Yes | 420 (55.05) | | 307 (40.24) | 113 (14.81) |  |
| No | 343 (44.95) | | 259 (33.94) | 84 (11.01) |  |
| family history of thyroid cancer (no/%) |  | |  |  | 0.017 |
| Yes | 29 (3.80) | | 16 (2.10) | 13 (1.70) |  |
| No | 734 (96.20) | | 550 (72.08) | 184 (24.12) |  |

**Table S2. Relationship between patient serological markers and thyroid nodules**

| **Characteristic** | **NO.** | **Benign** | **Malignant** | ***P* value** |
| --- | --- | --- | --- | --- |
| Liver function (U/L) |  |  |  |  |
| ALT | 24.73±17.76 | 24.25±15.85 | 25.53±20.11 | 0.565 |
| AST | 21.29±13.04 | 21.53±12.84 | 20.99±13.34 | 0.722 |
| Kidney function (µmol/L) | 67.01±29.22 | 65.33±12.97 | 69.73±44.28 | 0.145 |
| Lipid (mmol/L) |  |  |  |  |
| TC | 5.05±1.80 | 5.31±2.32 | 4.73±1.00 | 0.154 |
| TG | 1.78±1.56 | 1.95±1.65 | 1.57±0.99 | 0.382 |
| LDL | 2.87±0.99 | 2.93±1.01 | 2.70±0.95 | 0.259 |
| HDL | 1.52±0.44 | 1.59±0.45 | 1.25±0.27 | 0.111 |
| Blood glucose (mmol/L) | 5.56±1.26 | 5.63±1.17 | 5.46±1.39 | 0.209 |
| HbA1c (%) | 6.26±1.34 | 6.35±1.67 | 6.02±1.29 | 0.613 |
| Uric acid (µmol/L) | 307.76±84.85 | 304.47±76.17 | 313.01±98.38 | 0.327 |
| Thyroid function |  |  |  |  |
| FT3 (pmol/L) | 4.31±2.36 | 4.34±2.70 | 4.23±0.61 | 0.613 |
| FT4 (pmol/L) | 13.21±1.75 | 12.96±1.74 | 13.95±1.79 | 0.827 |
| TSH (mIU/L) | 1.91±1.24 | 1.89±1.17 | 1.95±1.43 | 0.657 |
| TSH (no./%) |  |  |  | 0.787 |
| < 1.2mIU/L | 254 (33.29) | 187 (24.51) | 67 (8.78) |  |
| 1.2-2.07mIU/L | 264 (34.60) | 195 (25.56) | 69 (9.04) |  |
| > 2.07mIU/L | 245 (32.11) | 184 (24.12) | 61 (7.99) |  |
| TGAB (no./%) |  |  |  | 0.491 |
| Negative | 488 (63.96) | 366 (47.97) | 122 (15.99) |  |
| Positive | 275 (36.04) | 200 (26.21) | 75 (9.83) |  |
| TPOAB (no./%) |  |  |  | 0.029 |
| Negative | 542 (71.04) | 414 (54.26) | 128 (16.78) |  |
| Positive | 221 (28.96) | 152 (19.92) | 69 (9.04) |  |
| TRAB (IU/mL) | 0.72±0.24 | 0.91±0.28 | 0.53±0.23 | 0.485 |
| TG (ng/mL) | 8.53 (3.07, 25.90) | 8.99 (3.24, 27.04) | 7.26 (2.03, 21.47) | 0.159 |
